# Carbon Ion Radiotherapy for Oligo-Recurrence in the Lung

**DOI:** 10.1155/2013/219746

**Published:** 2013-01-27

**Authors:** Naoyoshi Yamamoto, Mio Nakajima, Hirohiko Tsujii, Tadashi Kamada

**Affiliations:** Research Center for Charged Particle Therapy, National Institute of Radiological Sciences, Anagawa 4-9-1, Inage-ku, Chiba 263 8555, Japan

## Abstract

The clinical results after carbon ion radiotherapy for the metastatic lung tumors believed to be in the state of oligo-recurrence were evaluated. One hundred and sixteen lesions in 91 patients with lung cancer metastasis were treated with carbon ion radiotherapy at our institute from April 1997 to February 2011. Regarding the prescribed dose, total dose ranged between 40 gray equivalents (GyE) and 80 GyE, and fraction size ranged from 1 to 16 fractions. After a median followup period of 2.3 years (range, 0.3–13.1 years), the statistical overall survival rate and local control rate were 71.2% and 91.9% at 2 years after treatment, respectively. Treatment-related side effects were not a clinical problem. When classified by the primary organ, there were 49 cases of lung cancer, 20 cases of colorectal cancer, and 22 cases of others. The overall survival rate and local control rate for lung metastasis cases from lung cancer at 2 years after treatment were 81.5% and 92.4%, respectively, and 65.0% and 92.0% regarding lung metastasis from colorectal cancer. Carbon ion beam therapy for the metastatic lung tumors is a safe therapy, and the therapeutic effect is comparable to the outcome obtained from reported surgical resections.

## 1. Introduction

Radiotherapy is the principal treatment option for patients with early stage lung cancer and contraindications to receive surgery. The outcome from using conventional therapeutic techniques has been a 40–70% 5-year local control rate, but a local control rate equivalent to surgery is being reported due to recent advancements in irradiation techniques [1–4]. These irradiation techniques include SBRT, proton beam therapy, and carbon ion radiotherapy (CIRT).

Clinical trials for various types of tumors were initiated at the National Institute of Radiological Sciences (NIRS) from June 1994 using carbon ion beams, and dose fractionation suitable for individual diseases and irradiation techniques, such as a respiratory-gated radiotherapy and so forth, were developed. As a result, the healing of refractory cancers such as sarcoma of the bone and soft tissue, for which surgery is difficult, postoperative local recurrence of rectal cancer, and so forth, were achieved, and it was found that safe treatment is possible in a further shorter period regarding cancers of the prostate gland, the head and neck, lungs, and liver [1]. 

Treatment for nonsmall cell lung cancer was initiated in November 1994. Regarding peripheral stage I lung cancer, the fractionation number was gradually reduced from 9 times [5] to 4 times [6] while confirming the safety and efficacy. Currently, a clinical study is being carried out in which irradiation is completed in a day.

We herein report on our outcome from treating metastatic lung tumor believed to be in the state of oligo-recurrence [7], using carbon ion beams in which a good local control rate may be hoped for.

## 2. Materials and Methods

### 2.1. Patients

From April 1997 to February 2011, 116 lesions in 91 patients were treated with CIRT in our institute. The average age was 64.8 years old (range, 10–86 years) with a male/female ratio of 57/34. All patients were diagnosed by CT, PET, bone scintigraphy, and brain MRI before therapy. The histology and metastasis diagnosis of the tumors were determined based on the clinical course.

The conditions for applying the treatment to patients were as follows: the primary lesion is treated with no apparent local recurrence in the primary organ at the time of lung metastasis treatment, that is, the tumor is oligo-recurrence, there are no active lesions in organs other than the lungs, and there is one lesion in the lungs as a primary rule.

It is difficult to diagnose exactly the lung tumor as metastasis from primary lung cancer. In case, the lung tumor cannot be diagnosed as secondry primary lung cancer, we determined it as metastatic lung tumor. 

Regarding the number of lesions per patient treated with carbon ion therapy, 4 lesions were treated in 2 cases, 2 lesions were treated in 19 cases, and only one lesion was irradiated in 70 patients.

The prescribed dose ranged from 40 GyE to 80 GyE, and this was divided into several fractions. The fractionation regimen of 52.8 GyE in 4 fractions was the most commonly used for the treatment of the 116 lesions, which was used on 54 tumors. This was followed by 23 lesions of 60.0 GyE in 4 fractions. In many cases, 52.8 GyE in 4 fractions was used for lung metastasis from lung cancer while 60.0 GyE in 4 fractions was used for colorectal cancer.

When classified by the primary organ, there were 49 cases of lung cancer, 20 cases of colorectal cancer, and 22 cases of other cancers. The breakdown of organs classified as other cancers included various types such as bone and soft tissue tumors, cervical cancer, thymic cancer, esophageal cancer, pharyngeal cancer, ovarian cancer, pancreatic cancer, hepatic cancer, and breast cancer, with the number of cases according to these organs being 4 cases or less.

Regarding the major axis length of the lung tumor, a small tumor was considered to be 3 mm while a large tumor was 100 mm, with a median of 18 mm. The median length of the tumor according to the primary organ was 18 mm, 15 mm, and 19 mm, respectively, regarding lung metastasis from lung cancer, lung metastasis from colorectal cancer, and other types of lung metastasis.

The patient characteristics are provided in Table 1.

Past history comprising several elements such as age, pulmonary function, cardiac function, and so forth, as investigated regarding all patients, who were either diagnosed by a surgeon as being medically unsuitable for surgery due to coexisting diseases or the patients themselves did not wish to undergo surgery.

This study was approved by the institutional review board of NIRS and was conducted in accordance with the ethical standards provided by the Declaration of Helsinki. Informed consent was obtained from all patients prior to treatment. 

### 2.2. Treatment

Treatment was carried out within a week after treatment planning was created. In targeting, a visible legion on the CT image in the soft tissue condition was defined as the gross tumor volume (GTV). The clinical target volume (CTV) was determined by setting the margin more than 10 mm outside the GTV. To allow for the movement of the target during gated respiration, the internal margin was set by 5 mm outside the CTV. The planning target volume (PTV) was defined as CTV + internal margin. The total dose applied ranged from 40 GyE to 80 GyE to the isocenter, and 95% or more was irradiated to the PTV. Irradiation was carried out by dividing the total dosage into 1 to 16 fractions. Set-up corrections were carried out so that PTV would be less than 2 mm three dimensionally at every treatment.

### 2.3. Followup

Most patients underwent clinical examinations for followup, and CT scan of the thorax was carried out at our institute. Patients in which followup testing could not be carried out until completion underwent periodic CT scanning at another institute. The clinical outcomes of all patients have been confirmed.

The first followup examinations were performed 4 weeks after CIRT and in the following every 3 to 4 months. It is difficult to distinguish the change in normal tissues from radiation and tumor regrowth. We defined transitorily enlarged densities observed following approximately 3 months as locally controlled tumor. Meanwhile, local recurrence was determined from the enlarging tendency of tumors, as well as the outcome of CT image, PET scan, tumor marker, and biopsy.

## 3. Results

**Figure 1:**
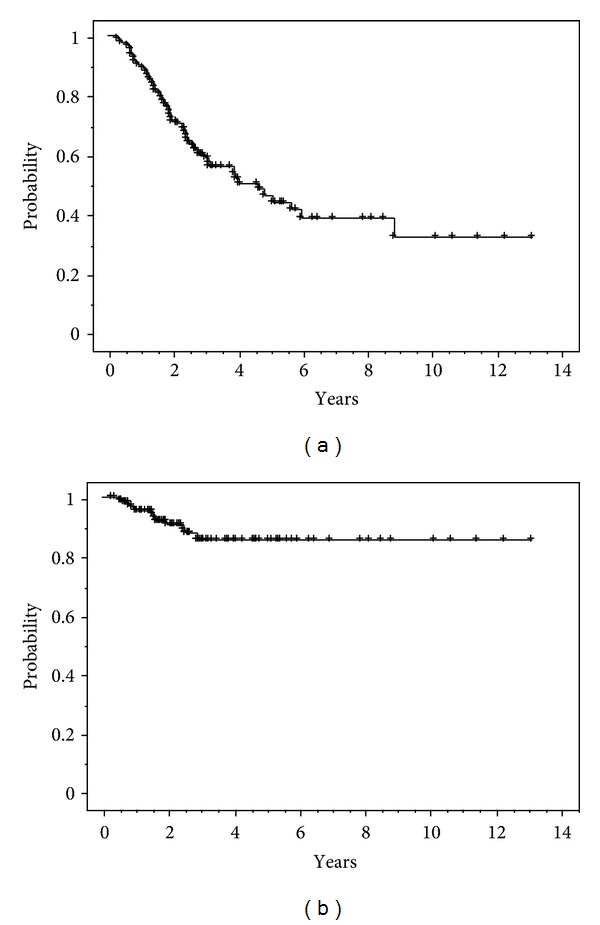
(a) Overall survival rate (*n* = 91). (b) Local control rate for lung metastases (*n* = 116).

The statistical 2-year overall survival rate of 91 patients was 71.2% with a median observation period of 2.3 years (range, 0.3–13.1 years, Figure 1(a)). The local control rate of the 116 treated lesions was 91.9% at 2 years after therapy (Figure 1(b)). 

The toxicities to the skin and lung caused by CIRT were assessed according to the NCI-CTC (early) and RTOG/EORTC (late). Early skin reactions were assessed for 116 lesions and late skin reactions for 114 lesions. Of the early reaction lesions, 116 were grade 1. Of the late reaction lesions, 114 were grade 1. Lung reactions were clinically assessed in the 116 lesions of 91 patients. Only five patients had grade 2 in early reaction; no adverse events greater than grade 2 were detected among early and late reactions. 

Twelve of 91 patients (12 of 116 lesions) had recurrences. In fifty-five of 91 patients, new lesions appeared in other sites, for example, lung, bone, and brain. In following this treatment, 47 patients died. Regarding the cause of death, 5 of 26 patients (19.2%) of all deceased lung cancer died due to causes other than the primary disease such as pneumonia and so on; however, in metastasis from other cancers such as colorectal cancer, the cause of death in all cases was cancer death due to the primary disease.

**Figure 2:**
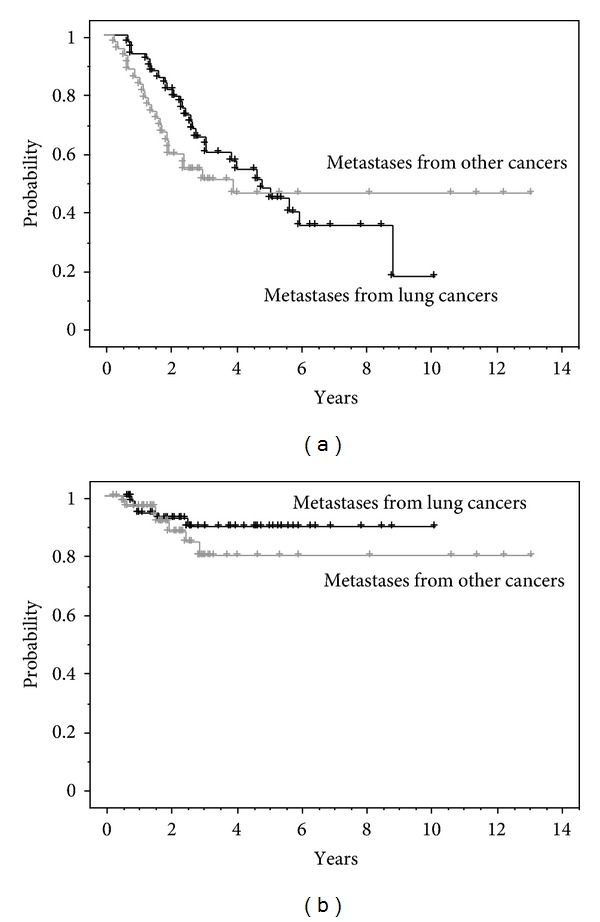
(a) Overall survival for lung metastases from lung cancer (*n* = 49) versus other cancer (*n* = 42). (b) Local control for lung metastases from lung cancers (*n* = 58) versus other cancers (*n* = 58).

The 2-year overall survival rate of lung metastasis cases from lung cancer was 81.5%, and the overall survival rate of lung metastasis from other than lung cancer was 59.3% (Figure 2(a)). The local control rate were 92.4% and 91.3%, respectively, (Figure 2(b)). Furthermore, the 2-year overall survival rate and local control rate of 30 lesions in 20 cases of lung metastasis from colorectal cancer was 65.0% and 92.0%. 

The survival rate of 70 cases with one lesion irradiated with carbon ion beams and 21 cases in which there were several irradiated regions was compared. The 2-year cause specific survival rate was 72.4% and 75.4%, with no significant difference (*P* = 0.3977).

**Figure 3:**
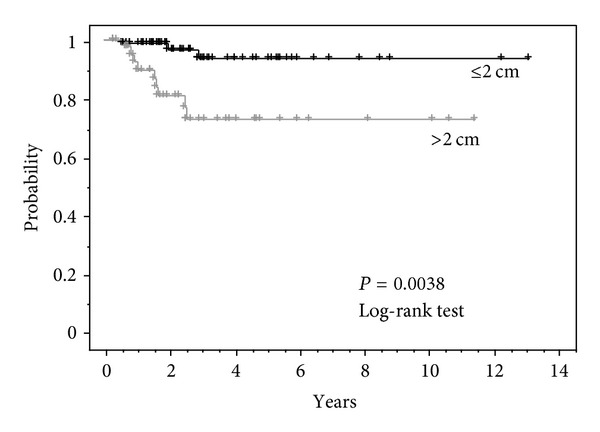
Local control for tumor diameter ≤2 cm (*n* = 72) versus >2 cm (*n* = 44) 3 y. Local control rate ≤2 cm: 93.4%, >2 cm: 72.7%.

The effect of the tumor size on local control was investigated. When the local control rate of 116 tumors was compared regarding the length of the tumor, the local control rate was significantly superior regarding those shorter than 2 cm compared to those exceeding 2 cm (Figure 3). When lung metastasis from lung cancer was compared in the same manner, the local control rate was 100% regarding tumors that are 2 cm or smaller in length.

**Figure 4:**
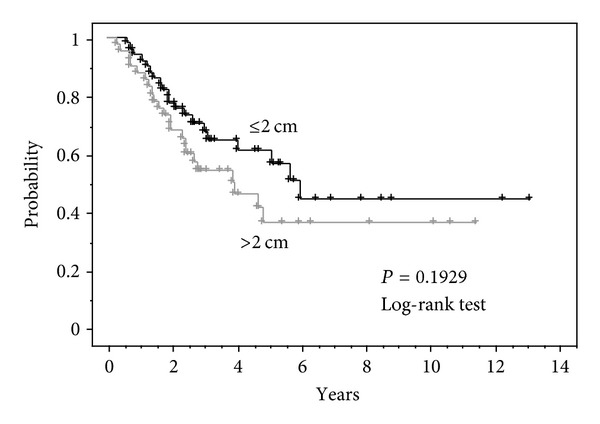
Cause specific survival for tumor diameter ≤2 cm (*n* = 50) versus >2 cm (*n* = 41) 3 y. Cause specific survival rate ≤2 cm: 70.6%, >2 cm: 54.3%.

Furthermore, the tumor size and the prognosis were investigated. The cause specific survival rate was compared regarding 50 cases in which the maximum diameter of the treated tumor was 2 cm or smaller and 41 cases in which it exceeded 2 cm. The 2-year cause specific survival rate was 77.5% regarding the group with a tumor diameter of 2 cm or smaller, and a good tendency was observed although there was no significant difference compared to those exceeding 2 cm, at 67.8% (*P* = 0.1929) (Figure 4).

Although there was no significant difference in comparing the cause specific survival rate of 10 cases in which local control was not obtained and other cases in which local control was obtained, there was no survivor of 5 years or longer regarding cases in which local control was not obtained.

The relationship between the time taken until commencing CIRT after treatment of the primary tumor and the prognosis was investigated in the cases of metastasis from lung cancer. The time taken until treatment of the primary lesion to treatment of the lung metastasis was classified into within 1 year, from 1 year to 2 years, from 2 years to 3 years, from 3 years to 5 years, and over 5 years, and the respective cause specific survival rates were compared. There was no difference in groups that took within 1 to 5 years until treatment, though the 3-year cause specific survival rate was from 60.6% to 72.7%. While in group of over 5 years, all 7 patients are still alive (median followup period: 3.5 years) except for one case of death due to another disease.

## 4. Discussion

We treated metastatic lung tumors believed to be “oligo-recurrence” using carbon ion beams in which a high local control rate may be expected. 

A diagnosis of metastasis was determined from the clinical course. There were many cases in which pathologic tissues were not sampled due to reasons such as the following: biopsy was difficult because the tumor was small, a malignant tumor was clearly suspected upon imaging and clinical course, diagnosis was obtained from resected lung tumors in the past by surgery. We believe that diagnosis with a malignant tumor was justifiable but discrimination with the primary lung cancer may be indicated as problematic, especially regarding cases diagnosed with lung metastasis from lung cancer. As mentioned below, this cannot be ruled out, although the possibility is low.

Regarding adverse reactions, there were no patients with grade 3 or more regarding both early-reaction NCI-CTC and late-reaction RTOG/EORTC. It is believed that the advantages of adopting respiratory-gated radiotherapy and irradiation from 4 directions are exhibited by the low frequency of normal tissue damage [8].

Considering the poor systematic medical condition of the patients, that is, the fact that many patients who are medically unsuitable for surgery are being treated and this is having a major effect on the outcome of overall survival, it was believed that the treatment outcome was generally good. The overall survival rate was favorably comparable to the outcome of CIRT for stage I lung cancer [5, 6], and we believe that this suggests that our criteria for selecting these cases was appropriate.

One lesion was determined as the subject as a general rule, but there were cases in which multiple lesions were treated as a result of clinical course. No difference was observed between the patients who treated single lesion and multiple lesions in comparison of survival. It cannot be determined that cases in which one location alone was irradiated ultimately had only one metastasis, and perhaps cases in which multiple lesions were treated were advantageous in that treatment was successfully completed.

The local control rate is discussed. It is believed that the local control rate is generally permissible. In this outcome, the local control rate for the tumors that are 2 cm or less was particularly superior. In contrast, the local control for the tumors exceeding 2 cm was by no means satisfactory compared to the outcome of the CIRT for primary lung cancer when considering that most tumors are 3 cm or smaller.

There are reports mentioning that tumor size is a prognostic factor [9]. In this study, we evaluated the overall survival concerning tumor size, not volume. Although the analysis outcome is omitted, smaller tumors have a tendency for better prognosis. One opinion is that the tumor doubling time affects the prognosis [10], thus suggesting that perhaps the same phenomenon is observed.

It is under discussion regarding whether or not the local treatment of metastatic lung tumors is effective for prolonging the prognosis. For the metastatic lesion, surgery or radiation therapy is carried out on some patients for potential effect, but the criteria for selecting cases in which an effect may be expected is not clear [11]. We determined the eligibility criteria as being the condition that is frequently used in carrying out surgical resection, that is, the primary lesion is controlled, there are no lesions in places other than the lungs, and there is only one lesion at the time of treatment as a rule. Whether or not our result is superior compared to chemotherapy and the best supportive care remains to be elucidated, but it was evaluated as being satisfactory compared to surgical resection cases due to metastasis [12] and the outcome of CIRT for stage I nonsmall cell lung cancer. In our outcome as well, there were no long-term survival cases of 5 years or more regarding cases in which local control was not achieved, although there was no significant difference, and it is believed that the prognosis is poor. However, in order to make an accurate evaluation, further analysis is necessary, including the effect of other treatments, such as chemotherapy, and the local control period.

There are reports mentioning that the period from treatment of the primary tumor is related to the prognosis after the treatment of lung metastasis [13]. The correlation between cause specific survival and the period from primary lesion treatment to metastasis treatment was investigated with lung metastasis cases from lung cancer in our cases as the subject; however, there was no clear difference. Moreover, 7 of 49 cases underwent the treatment of lung metastasis at 5 years or more after the primary lesion treatment; however, patients in all cases are still alive except for 1 case that died from another disease, so this group had good prognosis. Naturally, it cannot be denied that some primary lung cancers are mixed in from a diagnosis of metastasis.

From the results of this study, it was shown that high local control may be obtained by CIRT with suppressing adverse reactions and that an effect comparable to surgical resection may be obtained regarding metastatic lesions of a certain size. It is believed that an opportunity for treatment may be provided for oligo-recurrence cases in which resection could not be carried out in the past due to reasons such as declined pulmonary function. Furthermore, arguments that adaption may be expanded to cases that were not adaptable to treatment for multiple metastases in the past may be expected because CIRT is a low invasive remedy; however, it is believed that this must be carefully decided upon for evaluating whether or not a long-term prognosis is achieved.

In this report, the presence of metastatic lesions other than the lung or the treatment outcome thereof was not investigated. Regarding this, the treatment course of, for example, affiliated lymph node metastasis and brain metastasis in the case of lung cancer, and local lymph node metastasis, liver metastasis, in the case of colorectal cancer, must be analyzed and investigated in detail depending on the primary organ.

## 5. Conclusions

We herein reported on the outcome of CIRT for metastatic lung tumors diagnosed as being oligo-recurrent. Lung cancer occupied the majority regarding the breakdown of cases, with lung metastasis of colorectal cancer occupying half of the remaining cases. Lesions other than these were metastases of multiple types of cancer.

This is a safe and effective local treatment for lung metastasis patients without adaptation to surgery. Particularly regarding small tumors, the same tumor control as surgical treatment may be expected.

Considering the fact that many patients without medical adaptation to surgery are being treated, it is believed that the treatment outcome is good. And this indicates that our criteria for selecting oligo-recurrent cases were appropriate.

## Figures and Tables

**Table 1:** Patient characteristics.

Primary organ	Patient (*n*)	M/F	Age mean range	Tumor (*n*)	Size (mm) median range	Prescribed dose
Total	91	57/34	64.8	116	18	52.8 GyE/4 fr (*n* = 54)
			10–86		3–100	60.0 GyE/4 fr (*n* = 23)
						Others (*n* = 39)

Lung cancer	49	35/14	69.8	58	18	52.8 GyE/4 fr (*n* = 34)
			39–86		3–75	60.0 GyE/4 fr (*n* = 2)
						Others (*n* = 22)

Colorectal cancer	20	10/10	64.3	30	15	52.8 GyE/4 fr (*n* = 6)
			41–86		5–60	60.0 GyE/4 fr (*n* = 17)
						Others (*n* = 7)

Other cancer	22	12/10	49.1	28	19	52.8 GyE/4 fr (*n* = 14)
			10–84		7–100	60.0 GyE/4 fr (*n* = 4)
						Others (*n* = 10)
